# Evaluation of the Effect of Recycled Polypropylene as Fine Aggregate Replacement on the Strength Performance and Chloride Penetration of Mortars

**DOI:** 10.3390/polym14142806

**Published:** 2022-07-09

**Authors:** Fahed Alrshoudi, Ubair Abdus Samad, Othman Y. Alothman

**Affiliations:** 1 Department of Civil Engineering, College of Engineering, King Saud University, P.O. Box 800, Riyadh 12372, Saudi Arabia; falrshoudi@ksu.edu.sa; 2 Center of Excellence for Research in Engineering Materials (CEREM), King Saud University, P.O. Box 800, Riyadh 11421, Saudi Arabia; uabdussamad@ksu.edu.sa; 3 Department of Chemical Engineering, College of Engineering, King Saud University, P.O. Box 800, Riyadh 11421, Saudi Arabia

**Keywords:** recycled plastic, recycled polypropylene, cement mortar, aggregates, compressive strength, chloride penetration

## Abstract

Nowadays, the re-use and recycling of industrial wastes to reduce the environmental impact and landfill problems are the main concerns of researchers. Plastics are one of the main waste materials worldwide, with considerable impacts on health and environmental conditions. Recycling plastic wastes in the concrete industry is one of the adopted ways to reduce such impact and increase the economic recyclability of plastics. In this study, the utilization of recycled polypropylene (rPP) as a fine aggregate in the preparation of cement mortars was evaluated. The river sand was replaced with 10, 20, 30, 40, and 50%, volumes of rPP. The results showed that the inclusion of rPP reduced the mortar’s workability and fresh density. Fresh density dropped from 11% to 35% as the rPP content increased. Furthermore, the compressive strength at early and late age was significantly influenced by the rPP content. At 28 days of curing age, the results showed that the inclusion of 50% of rPP in the mortar matrix led to a drop in the compression strength from 40 MPa to 10 MPa. A similar trend of results was obtained for the flexural (from 8.3 MPa to 2.9 MPa) and tensile strengths (from 3.4 MPa to 1.21 MPa). The chloride ion penetration went through a maximum of 5000 Coulombs between 10% and 50 % of rPP. Therefore, it can be concluded that the use of 10% of rPP as a river sand replacement can achieve acceptable strength (25 MPa) for several applications in the construction industry.

## 1. Introduction

With the beginning of a new industrialization era, the world is encountering environmental issues related to the high production of industrial wastes. A significant increase in the use of plastic products globally has been witnessed in the past few decades. According to a report, the production of plastic materials in 2019 was approximated 3.7 × 10^8^ metric tons, which is on a continuous yearly rise [1]. In the majority of the cases, plastic materials are designed to be used for disposable applications, such as packaging materials, which is the prime cause of waste generation [2]. The accumulation of plastic waste is of greater concern, as it is the main cause of some environmental and health issues. It has been estimated that, globally, 192 countries on the coastal belt produced 2.75 × 10^8^ metric tons of waste in 2010, out of which approximately 1.7% to 4.6% was dumped into the ocean [3]. The majority of this ocean-disposed waste degrades, settles below the surface of the sea, and becomes a part of marine organisms, damaging the ecosystem [4], and which could possess undesired effects on the ecological environment and human health [5,6].

The re-use of plastics, particularly in construction, is gaining momentum, because it can potentially provide a solution to minimize environmental pollution by preventing plastic waste being dumped in landfills and incinerated. Studies suggest that plastic waste can be used in concrete as a partial substitute for sand and coarse aggregates, such as polyethylene terephthalate (PET) [7,8,9,10,11,12], polystyrene (PS) [13,14], polyvinyl chloride (PVC) [15,16,17,18,19], high-density polyethylene (HDPE) [20,21,22,23], low-density polyethylene (LDPE) [24,25,26,27], and blends of different polymers [28,29,30,31].

Different researchers have studied different factors affecting the addition of polymer-filled mortars, including polymer content [16,20,21,24,27]; curing conditions, including time, temperature, and media [28,31,32]; mortar porosity, and cement type [16]. Thiam et al. [28] used waste plastic as a binder and investigated the curing time and media, and found that air curing resulted in higher mechanical strength than water curing due to slower cooling, which allowed for a higher rate of crystallization. Moreover, increasing the curing time increased the compressive strength. Amin et al. [32] found that increasing the cure temperature increased the compression strength in most cases, probably as a result of the hydration reaction increase.

The addition of plastic waste as a replacement produces different outcomes depending on the percentage of the replacement. In all the studies, it has been concluded that, with the addition of plastic waste, the unit weight declines significantly when increasing the replacement [33,34,35,36]. Moreover, many studies also suggest a decline in flexural, compressive, and splitting tensile strengths [25,37,38]. Compressive strength has been reported to decrease by increasing the recycled plastics. Suganthy et al. [20] reported a decrease in compressive strength by 40.2% by increasing the HDPE content in the aggregate from 0% to 50 %, and a further decrease of 22.3% when HDPE was increased 100%. Similarly, Badache et al. [21] reported a linear drop in compressive strength of 57% when increasing recycled HDPE up to 60%. Shanmugapriya and Santhi [23] found that the compressive strength dropped approximately 25% when adding 20% HDPE waste. A similar trend was reported for recycled LDPE [24,26,27].

In the application of polymer concrete, Yu et al. [39] incorporated crumb rubber and short fibers of polypropylene, along with glass waste fibers and crumb rubber in epoxy composites, to improve the mechanical performance of the composites.

Recently the use of recycled polymers in geopolymer concrete has been under investigation [40]. Abousnina et al. [41] added short fibers, including polypropylene fibers, to enhance the mechanical properties of geopolymer mortar containing fine oil-contaminated sand. They found that fiber addition improved the strength of the geopolymer mortar. Posi et al. [42] reported the enhancement of the properties of geopolymer concrete, such as absorption and thermal insulation with acceptable strength, by adding recycled plastics as aggregates. 

The overall decrease in mechanical properties of plastic-filled mortar can be attributed to plastic properties, such as low elasticity and large molecules compared to sand aggregate. It also could be due to weak adhesion between plastics and cement, as plastics are hydrophobic [27,43]. Another reason might be the formation of stress concentration due to voids or air [29,44]. 

Although some properties of mortars are generally affected by plastic addition, studies emphasized that adding recycled plastics to mortars or concrete could assist some properties at certain levels to be used in non-engineering applications, while reducing plastic disposal and helping its sustainability [22,27,31,45]. Ferreira et al. [31] suggested that a 10–15% replacement of normal aggregates with plastic waste could maintain acceptable mechanical properties.

Most literature studies deal with PET and PE, with less focus on PP aggregate replacements; this paper aims to contribute to the studies of PP replacement. Thus, this work’s goal is to evaluate the effect of rPP as a fine aggregate replacement on fresh and hardened mortar properties, and obtain the optimum level of replacement to achieve the acceptable strength value. For this purpose, several tests are adopted, such as flow diameter, fresh density, compressive, splitting tensile, and flexural strengths, and chloride penetration. The next step is to extend this work to examine the effect of the local weather on concrete mixed with PP. This should benefit the local building and construction industry. It also should encourage the utilization of plastic waste.

## 2. Materials and Methods

In this study, ordinary Portland cement (OPC), satisfying the ASTM C150 requirement for type I cement, was used to prepare the mortar specimens. The OPC was obtained from a local cement producer in Saudi Arabia and utilized as the primary source of calcium oxide. The rPP was purchased from the local market, with a particle size of no more than 4 mm and used as a fine aggregate to replace river sand.

**Figure 1:**
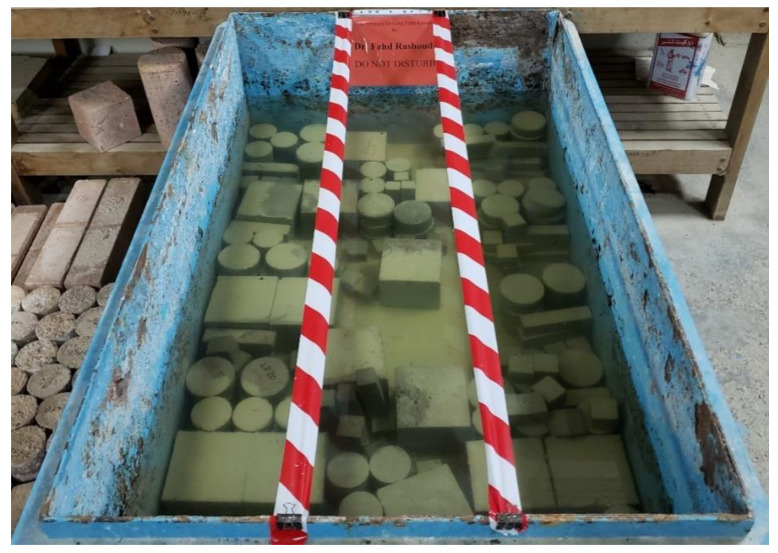
Curing condition of proposed mortar specimens.

In preparing the mortar specimens, the mix design was very important in order to produce a batch of mortar with desirable properties. For the purpose of this study, mortar specimens as control samples were prepared using a cement to fine aggregate ratio (c/a) of 1:3 and cement to water ratio (c/w) of 2:1. As shown in Table 1, the content of OPC, fine aggregates, and w/c ratios were fixed for all mixtures; then, the river sand was replaced with 10, 20, 30, 40, and 50% of recycled plastic. To prepare the suggested mortar specimens, river sand and recycled plastic were mixed for a duration of three minutes in dry conditions, which were then further mixed with OPC for a period of four minutes. The resultant product was then activated by adding tap water, and then blended in a machine for a further five minutes at medium velocity. The final step in the process was the introduction of the resulting mortar into molds, which was achieved by using a two-layer pouring method. In this process, each layer was subjected to vibration for a period of 15 s in order to eliminate any air pockets within the mixture. Once the casting process was completed, the mortar specimens were cured for 3 days in water (as shown in Figure 1); then, the specimens were left in lab conditions till the test date. 

**Figure 2:**
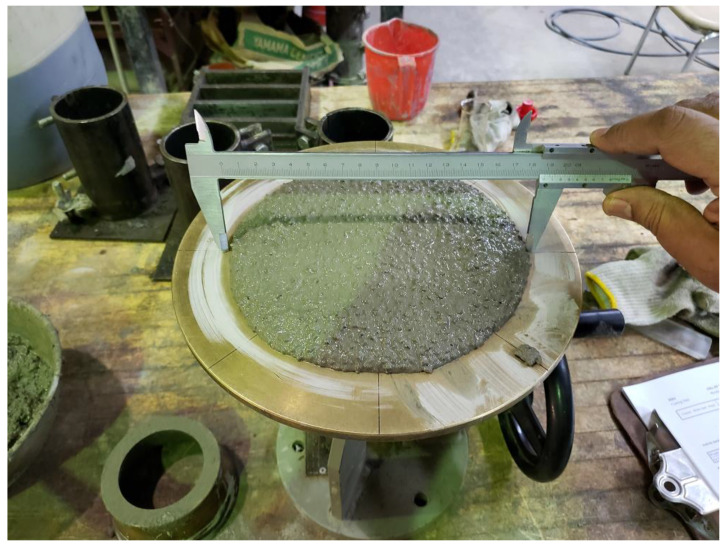
Evaluation of the workability performance of prepared mortars.

In this work, the effect of rPP content on fresh and hardened performance of proposed mortars was evaluated in terms of flowability, density, compressive, flexural, and splitting tensile strength, and chloride ion penetration. The flowability of the proposed mortars was measured using a flow table method modified from ASTM C230 (Standard Specification for Flow Table for Use in Tests of Hydraulic Cement). The flow table provides an efficient means of determining the flow of cement pastes and hydraulic cement mortars. A specific volume of material was melted on the table using the included flow mold. The mold was then removed, and the table was subjected to a specific number of 1/2 in. (12.7 mm) drops using the crank handle. The increase in the average diameter of the sample indicated the flow. Figure 2 shows the method of measuring the flow of the prepared mortars.

The compressive strengths (CSs) of the mortars were measured using ASTM C109/C109M-20b. Three mortar cubes for each category were prepared using steel molds of 50.8 mm a side (2-inch sides). The CSs of mortars mainly depended on a number of factors, which included the properties and proportions of the constituent materials, degree of hydration, rate of loading, method of testing, and specimen geometry. The properties of the constituent materials mainly affected the strength. The CS test method consisted of applying a compressive axial load to molded mortar cubes at a continuous rate without shock and within a prescribed range until failure occurred. Testing variables had a considerable influence on the measured compressive strength. Following the test procedures, the specimens were left to cure for 7, 28, and 56 days prior to the test. The cubes were placed in the testing machine to ensure that they were directly under the center of both the top and bottom plates. They were then loaded at a rate of 1.4 MPa per minute until failure. The failure load was recorded, and the cube was removed in preparation for the next test. The compressive strength of each cube was calculated as follows:

σ = P/A(1)

where σ is the ultimate compressive strength (MPa); P is the maximum applied load (kN); A is the cross-sectional area of the specimen (mm).

The tensile strength of mortar is a fundamental material characteristic used to predict crack formation. This property is generally expressed as a function of the compressive strength of a material. The splitting tensile strength of mortar is typically determined with a split cylinder test in accordance with ASTM C348-21. The tensile strength affects other properties, such as stiffness, damping action, bond to embedded steel, and the durability of the mortar. The tensile strength is determined either with a direct tensile test or with an indirect tensile test, such as a flexural or split-cylinder test. It is often assumed that the direct tensile strength of a mortar is approximately 10% of its compressive strength. The diameters of the specimens were first marked using a pencil prior to the test. Then, the specimen was placed on its side on top of a bearing strip of plywood with a nominal thickness of 1/8 inches and an approximate width of 1 inch. The length of the bearing strip had to be the length of the specimen or slightly longer. Another bearing strip was placed on top of the cylinder. The cylinder was then loaded at a constant rate that ranged from 0.68 to 1.3 MPa per minute. The cylinder was tested to failure and the ultimate load was recorded. For each category, three cylinders were tested. The splitting tensile strength of a cylinder was calculated using the following equation:

T = (2P)/(πLD)(2)

where T is the ultimate spitting tensile strength (MPa); P is the maximum applied load indicated by the testing machine (kN); L is the length of the specimen (mm); D is the diameter of the specimen (mm).

Flexural strength is the ability of a beam to resist failure in bending. The flexural strength is expressed as the modulus of rupture in MPa. The flexural strength is approximately 12 to 20% of the compressive strength. In the test, the three specimens for each category were subjected to concentrated loads at a third point in order to produce a pure bending movement. The test was conducted in accordance with ASTM C78. In the central-point loading, a beam of span L was loaded with a concentrated load P at the mid-span of the beam. The beam was supported as a simple beam. The deflection at the center of the span was:

Δ = PL^3^/48EI (3)

where L is the clear span at supports (mm); P is the concentrated load (N); E is the modulus of elasticity (GPa); I is the moment of inertia about the centroidal axis; Δ is the mid-span deflection (mm).

The chloride ion penetration test was conducted to study the effect of chloride ions in mortar specimens. This test was conducted at 28 days of curing age. The test for measuring the chloride diffusion was carried out using a cylindrical mortar specimen with a diameter of 100 and 200 mm thickness. This test was conducted following ASTM C 1202-97. Three samples were tested for each category.

The optical microscopy technique was used to investigate the structure of the rPP- filled mortar. An optical microscope (Stereomicroscope SteREO Discovery.V12 with motor focus, SYCOP control, and coaxial incident-light brightfield illuminator) was used, with magnification of ×8. Three-centimeter cube samples were cut by a special cutter to be used under the microscope.

## 3. Results and Discussion

### 3.1. Fresh Unit Weight

**Figure 3:**
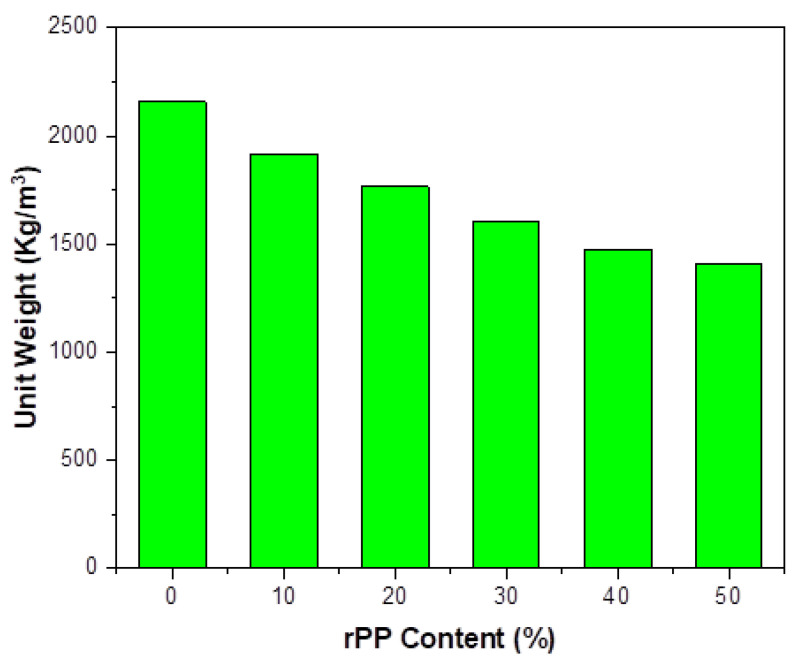
rPP addition effect on fresh unit weight of concrete.

The replacement of aggregate with rPP tended to lower the overall unit weight, as shown in Figure 3. The low density of rPP tended to decrease the density of the concrete, resulting in lighter concrete. The obtained results indicated that concrete without any replacement showed a unit weight of 2158 kg/m^3^. The replacement of aggregates with 10%, 20%, 30%, 40%, and 50% of rPP resulted in a decrease in unit weight, which was found to be 1916 kg/m^3^ (11%), 1786 kg/m^3^ (17%), 1608 kg/m^3^ (25%), 1478 kg/m^3^ (31%), and 1408 kg/m^3^ (35%), respectively. This drop in unit weight was because of the addition of rPP, which has a low bulk density and was thereby responsible for a decrease in the overall weight, which was in accordance with the results published by other researchers [9,31,45].

### 3.2. Dry Unit Weight

**Figure 4:**
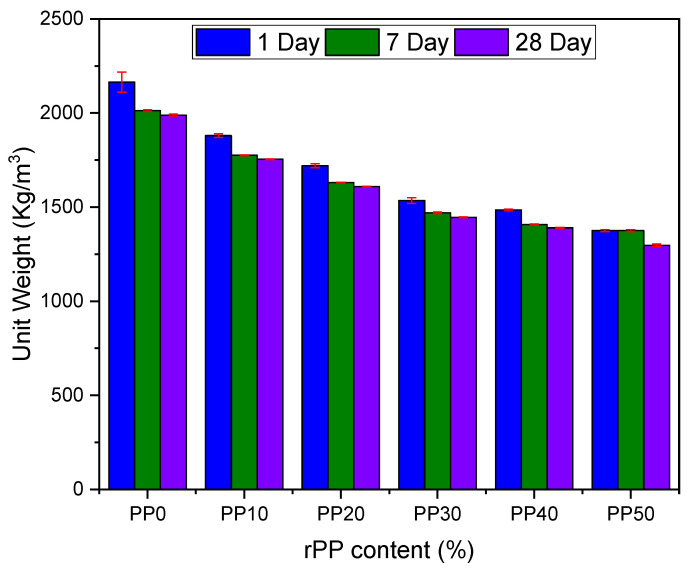
Effect of rPP addition on dry unit weight at different ages.

Figure 4 represents the dry unit weight of reference and different ratios of rPP mix samples at 1, 7, and 28 day intervals. It can be observed that with the increasing rPP content, the dry unit weight of concrete decreased. At the age of 28 days, the unit weight of the reference sample was 1989 kg/m^3^, while the unit weight of the sample with 30%, 40%, and 50% rPP replacement was found to be 1446 kg/m^3^ (27.30%), 1390 kg/m^3^ (30.11%), and 1297 kg/m^3^ (34.79%), respectively. The decrease in the unit weight of the same sample at intervals of 7 and 28 days was not significant. The decreasing weight with increasing rPP percentage was because of the lighter density of rPP plastics in comparison to the natural aggregates used in the reference sample. The results were in accordance with the findings of other researchers [10,19,33].

### 3.3. Compressive Strength

**Figure 5:**
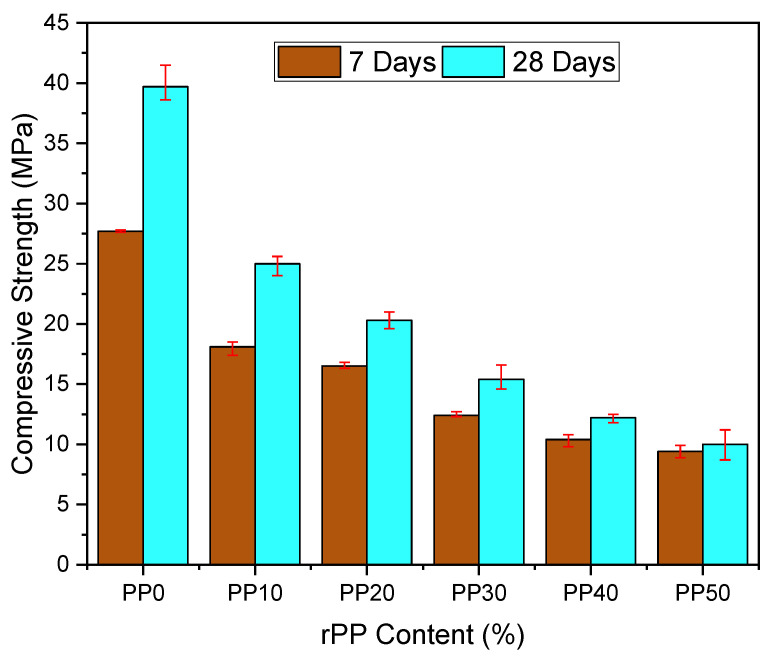
Compressive strength of mortar at 7 and 28 days.

**Figure 6:**
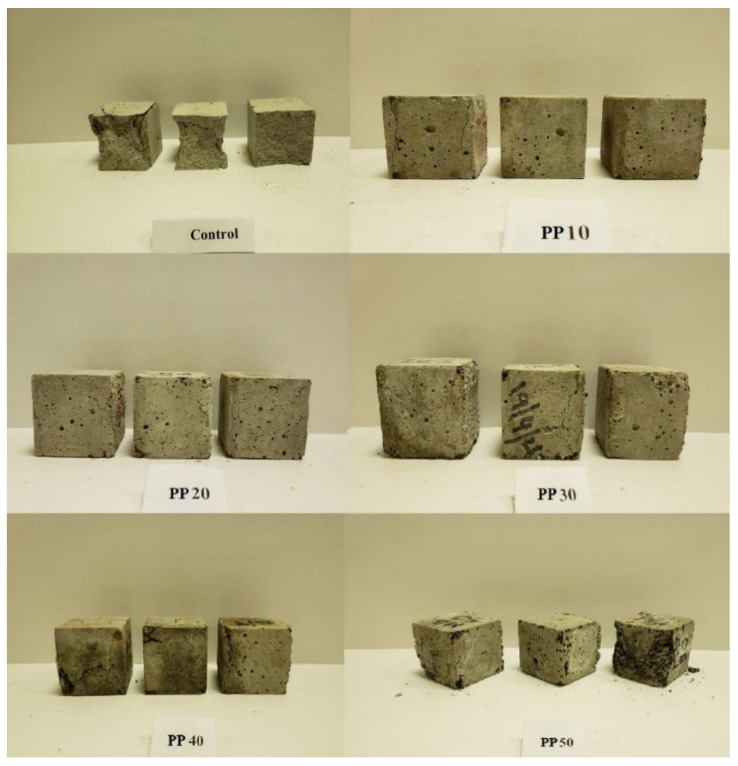
Failure modes of the proposed mortars containing various contents of rPP as natural aggregate replacement.

With the addition of rPP in various percentages, the compressive strength of the prepared concrete was measured at the age of 7 days and 28 days, as shown in Figure 5. The compressive strengths for the reference samples were 27.7 and 39.7 MPa for 7 and 28 days, respectively. The compressive strength of the concrete mix decreased by 61.2%, 69.2%, and 74.8% for the 30%, 40%, and 50% rPP mixes, respectively. The decrease in the compression strength was mainly because of the loss of adhesion between the cement and rPP, which resulted in the formation of voids [11,14,33,38]. Additionally, this decrease in compression could be attributed to the reduction in the composites’ bulk density of mortar as a result of increasing the PP. The failure modes for the cubic specimens of the prepared mortar containing various contents of rPP as the fine aggregate replacement under the static load are illustrated in Figure 6. The prepared mortar specimens with various contents of rPP (10-50%) were still in good shape at the ultimate compressive load-bearing conditions, while the specimens for the natural fine aggregates were sharply chipped. This could be attributed to the change in the transition zone, where the failure mode changed from a sudden brittle mode to a more ductile mode. However, more investigation is needed. The results also ensured that the compressive strength increased with curing time. However, the impact of curing time decreased as the rPP content increased. 

### 3.4. Flexural Strength

**Figure 7:**
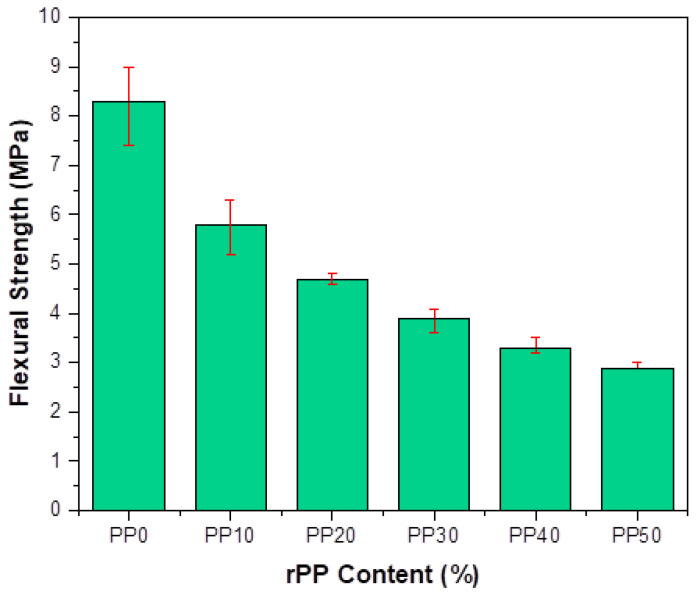
Flexural strength with different percentages of rPP in mortar at the age of 28 days.

The flexural strength for the prepared concrete with different percentages of rPP is shown in Figure 7. The observed effect of the flexural strength was similar to the compression strength, where more than a 50% decline in flexural strength was recorded for concrete mixture above a 30% replacement ratio. The flexural strength values obtained for the reference sample and concrete mix with a 30%, 40%, and 50% replacement were 8.3 MPa, 3.9 MPa, 3.3 MPa, and 2.9MPa, which represented a decline of 53%, 60.2%, and 65% in comparison to the reference sample. This reduction in flexural strength was because of the added rPP aggregates. The load-bearing capability of rPP aggregates was far less compared to the natural aggregates. Another factor that contributed to the reduction in properties with a higher percentage of replacement was low bond strength. The bonding between the plastic aggregates and cement was lesser because of its hydrophobic nature, which was the main factor for the loss of adhesion. Similar results are reported in the literature, where different types of plastic waste caused a reduction in flexural strength with an increasing plastic waste ratio [19,30].

### 3.5. Splitting Tensile Strength

**Figure 8:**
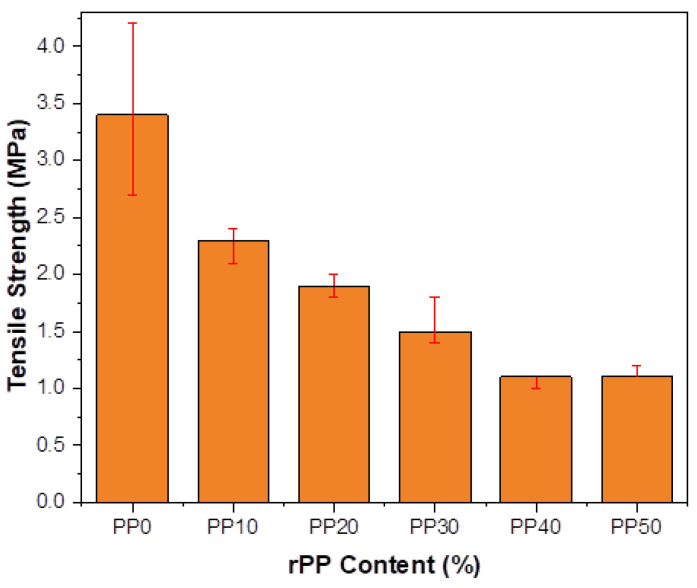
Tensile strength with different percentages of PP waste in mortar at the age of 28 days.

**Figure 9:**
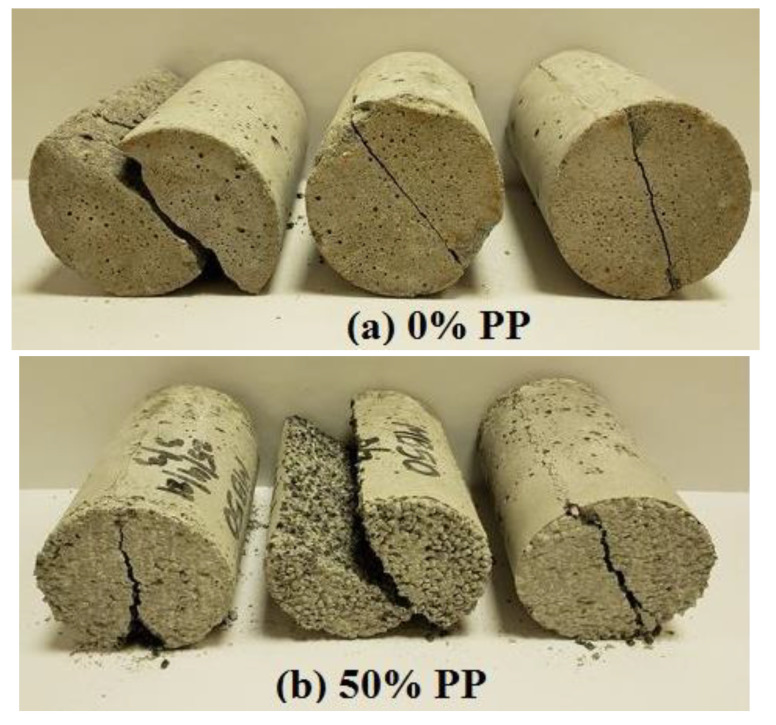
Effect of PP content on failure mode of tested specimens under splitting tensile load.

Splitting tensile strength was used to determine the tensile strength of concrete using a cylindrical shape specimen, which split across the vertical diameter. At the age of 28 days, the prepared concrete samples were subjected to a splitting tensile test. The results are shown in Figure 8. The effect of replacing aggregates with PPs on tensile strength had similar behavior to compression and flexural strength. It can be seen that replacing more aggregates with rPP led to a decrease in the tensile strength. In comparison to the controlled sample, the tensile strength of the concrete mix with a 50% replacement reduced from 3.4 to 1.1 (67.6%). The decrease in properties with the addition of plastic was due to plastic’s inability to absorb water. Additionally, the smoothness of the plastic surface caused a weakness in the bond between cement and plastic. Therefore, replacing more aggregates with plastic waste resulted in weak adhesion between the plastic and cement paste [17,43]. Other researchers suggested a zone formation and void (or air) content, where stress is concentrated, leading to failure [16,29,44]. Merlo et al. [16] suggested that plastic fillers, due to their relatively low mechanical properties and poor adhesion, behave as porosities in the aggregate, which weakens the mortar matrix. Figure 9 shows the failure mode of the mortar specimens containing a high volume of rPP (50%) compared to the control sample. It can be seen that the mode of failure between the control and 50% specimens was different, which could be as a result of the bonding effect between the polymer and the cement. However, more investigation is needed.

### 3.6. Optical Microscopy

**Figure 10:**
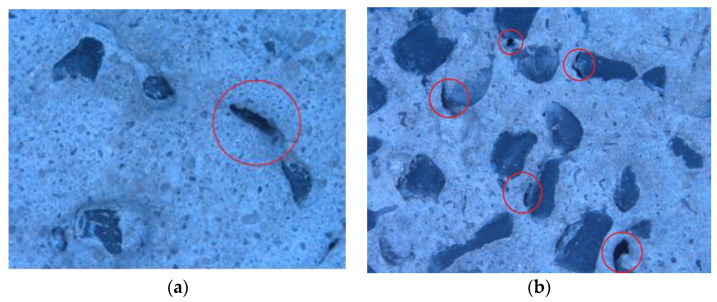
Optical microscope images of the prepared samples (**a**) with 20% rPP replacement and (**b**) with 50% rPP replacement.

In order to analyze the mixing of rPP with concrete, an optical microscopy analysis was performed on samples with 20% and 50% rPP, as shown in Figure 10.

Samples with 20% rPP possessed clear voids or air pockets around the rPP pellets (Figure 10a). With an increase in the percentage of rPP to 50%, these voids became more pronounced and even cracks were also clearly visible (Figure 10b). This suggests the poor strength and load-bearing capability of the prepared concrete, as witnessed through the decreasing tensile, flexural, and compressive strength. These visible voids in the samples acted as failure sites from where the degradation of the material started, ultimately reducing the overall performance of the material.

### 3.7. Chloride Ion Penetrability

**Figure 11:**
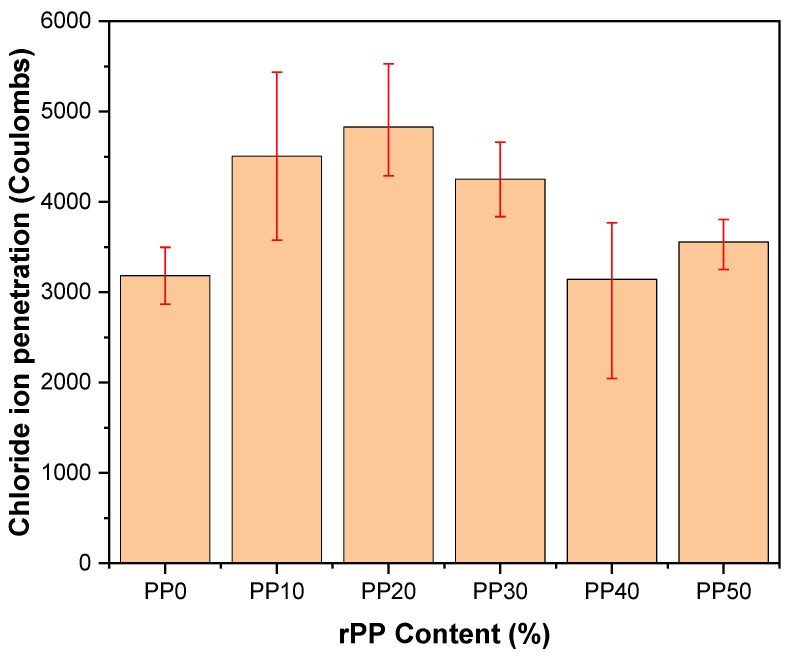
Penetration of chloride ions in mortar mix at the age of 28 days.

The penetration of chloride ions in concrete is influenced by factors such as the absorption of water, the degree of porosity, and concrete’s water permeability, as a more compact structure results in a lesser chloride ion penetration. The addition of plastic waste to concrete caused it to be prone to void formation due to the lack of adhesion between the concrete and plastic waste. The graphical presentation of the results obtained from concrete prepared with different percentages of plastic waste is shown in Figure 11 below. It can be seen that at lower percentages of rPP, the penetration of chloride ions was higher, while at higher rPP, the penetration of chloride ions was somewhat similar to the controlled sample. It has been reported in the literature that the addition of plastic waste does not necessarily increase the chloride ion penetration, as the presence of plastic granules acts as a blockage in the path of chloride ions [6]. The results were in accordance with findings in the literature [15,18]. Here, the penetration of chloride ions tended to reach maximum at 20% rPP, and then decreased to level off with the penetration of the control sample.

## 4. Conclusions

In this work, the possibility of producing sustainable construction materials using waste plastics, such as rPP, as a river sand replacement was explored. From the obtained results, it can be concluded that replacing more aggregates with rPP led to a higher reduction in mechanical properties. The following conclusions were derived:

- The replacement of natural fine aggregates with rPP led to a reduction in the workability of the prepared mortars.
- The fresh and hard unit weight of the proposed mortars tended to decrease with an increasing rPP content in the mortar matrix as a fine aggregate replacement. At a 50% replacement, the loss in the unit weight was approximately 35%.
- Compressive strength of the prepared mortar was negatively influenced by the rPP content. The results showed that the mortar prepared with 50% of rPP aggregates lost 75% of its strength. However, the specimens containing 10% rPP showed acceptable compressive strength for the construction industry.
- The reduction in the flexural strength was up to 65% at a 50% replacement.
- A similar trend of results was observed for flexural and splitting tensile strengths, and the strength values tended to decrease with an increasing rPP content. Due to a 50% of replacement, the reduction in the tensile strength was 67.6%.
- The durability of the mortar was significantly influenced by the rPP content. The chloride ion penetration value tended to increase with an increasing rPP content.
- Overall, the recycled-plastic-incorporated mortars showed a good potential for specific non-bearing lightweight construction applications while reducing the environmental impacts of plastic wastes. For example, they could be used in pavements and non-bearing loading walls.

## Figures and Tables

**Table 1:** Mortar mix design of rPP as a fine aggregate replacement (c/a = 1:3 and c/w = 2:1).

Mix	Fine Aggregates		rPP w% in Aggregates
	River Sand	PP Fine Aggregates	
PP0	3.0	0.0	0
PP10	2.70	0.30	10
PP20	2.40	0.60	20
PP30	2.10	0.90	30
PP40	1.80	1.20	40
PP50	1.50	1.50	50

## Data Availability

Not applicable.

## References

[1] Tiseo I. (2022). Production of Plastics Worldwide from 1950 to 2020. https://www.statista.com/statistics/282732/global-production-of-plastics-since-1950/.

[2] PlasticsEurope (2020). Plastics—The Facts 2020: An Analysis of European Plastics Production, Demand and Waste Data.

[3] Jambeck J.R., Geyer R., Wilcox C., Siegler T.R., Perryman M., Andrady A., Narayan R., Law K.L. (2015). Plastic waste inputs from land into the ocean. Science.

[4] Lebreton L., Egger M., Slat B. (2019). A global mass budget for positively buoyant macroplastic debris in the ocean. Sci. Rep..

[5] Rai P.K., Lee J., Brown R.J., Kim K.-H. (2021). Micro- and nano-plastic pollution: Behavior, microbial ecology, and remediation technologies. J. Clean. Prod..

[6] Almeshal I., Tayeh B.A., Alyousef R., Alabduljabbar H., Mustafa Mohamed A., Alaskar A. (2020). Use of recycled plastic as fine aggregate in cementitious composites: A review. Constr. Build. Mater..

[7] Albano C., Camacho N., Hernández M., Matheus A., Gutiérrez A. (2009). Influence of content and particle size of waste pet bottles on concrete behavior at different w/c ratios. Waste Manag..

[8] Choi Y.W., Moon D.-J., Chung J.-S., Cho S.-K. (2005). Effects of waste PET bottles aggregate on the properties of concrete. Cem. Concr. Res..

[9] Islam M.J., Meherier M.S., Islam A.K.M.R. (2016). Effects of waste PET as coarse aggregate on the fresh and harden properties of concrete. Constr. Build. Mater..

[10] Azhdarpour A.M., Nikoudel M.R., Taheri M. (2016). The effect of using polyethylene terephthalate particles on physical and strength-related properties of concrete; a laboratory evaluation. Constr. Build. Mater..

[11] Juki M.I., Awang M., Annas M.M.K., Boon K.H., Othman N., Roslan M.A., Khalid F.S. (2013). Relationship between compressive, splitting tensile and flexural strength of concrete containing granulated waste Polyethylene Terephthalate (PET) bottles as fine aggregate. Adv. Mater. Res..

[12] Araghi H.J., Nikbin I., Reskati S.R., Rahmani E., Allahyari H. (2015). An experimental investigation on the erosion resistance of concrete containing various PET particles percentages against sulfuric acid attack. Constr. Build. Mater..

[13] Tang W.C., Lo Y., Nadeem A. (2008). Mechanical and drying shrinkage properties of structural-graded polystyrene aggregate concrete. Cem. Concr. Compos..

[14] Wang R., Meyer C. (2012). Performance of cement mortar made with recycled high impact polystyrene. Cem. Concr. Compos..

[15] Kou S.C., Lee G., Poon C.S., Lai W.-L. (2009). Properties of lightweight aggregate concrete prepared with PVC granules derived from scraped PVC pipes. Waste Manag..

[16] Merlo A., Lavagna L., Suarez-Riera D., Pavese M. (2020). Mechanical properties of mortar containing waste plastic (PVC) as aggregate partial replacement. Case Stud. Constr. Mater..

[17] Mohammed A.A., Mohammed I.I., Mohammed S.A. (2019). Some properties of concrete with plastic aggregate derived from shredded PVC sheets. Constr. Build. Mater..

[18] Senhadji Y., Escadeillas G., Benosman A.S., Mouli M., Khelafi H., Kaci S.O. (2015). Effect of incorporating PVC waste as aggregate on the physical, mechanical, and chloride ion penetration behavior of concrete. J. Adhes. Sci. Technol..

[19] Aciu C., Ilutiu-Varvara D.-A., Manea D.-L., Orban Y.-A., Babota F. (2018). Recycling of plastic waste materials in the composition of ecological mortars. Procedia Manuf..

[20] Suganthy P., Chandrasekar D., Kumar S. (2013). Utilization of Pulverized Plastic in Cement Concrete as Fine Aggregate. Int. J. Res. Eng. Technol..

[21] Badache A., Benosman A.S., Senhadji Y., Mouli M. (2018). Thermo-physical and mechanical characteristics of sand-based lightweight composite mortars with recycled high-density polyethylene (HDPE). Constr. Build. Mater..

[22] Tamrin, Nurdiana J. (2021). The Effect of Recycled HDPE Plastic Additions on Concrete Performance. Recycling.

[23] Shanmugapriya M., Santhi H. (2017). Strength and chloride permeable properties of concrete with high density polyethylene waste. Int. J. Chem. Sci..

[24] Chaudhary M., Srivastava V., Agarwal V. (2014). Effect of waste low density polyethylene on mechanical properties of concrete. J. Acad. Ind. Res..

[25] Ohemeng E.A., Ekolu S.O. (2019). Strength prediction model for cement mortar made with waste LDPE plastic as fine aggregate. J. Sustain. Cem.-Based Mater..

[26] İpek S., Diri A., Mermerdaş K. (2021). Recycling the low-density polyethylene pellets in the pervious concrete production. J. Mater. Cycles Waste Manag..

[27] Mohammed A., Ali T.K.M., Rajab N., Hilal N. (2020). Mechanical properties of concrete and mortar containing low density polyethylene waste particles as fine aggregate. J. Mater. Eng. Struct..

[28] Thiam M., Fall M., Diarra M.S. (2021). Mechanical properties of a mortar with melted plastic waste as the only binder: Influence of material composition and curing regime, and application in Bamako. Case Stud. Constr. Mater..

[29] Vaccaro P.A., Galvín A.P., Ayuso J., Barbudo A., López-Uceda A. (2021). Mechanical performance of concrete made with the addition of recycled macro plastic fibres. Appl. Sci..

[30] Ruiz-Herrero J.L., Nieto D.V., Lopez-Gil A., Arranz A., Fernández A., Lorenzana A., Merino S., De Saja J.A., Perez M.A.R. (2016). Mechanical and thermal performance of concrete and mortar cellular materials containing plastic waste. Constr. Build. Mater..

[31] Ferreira L., de Brito J., Saikia N. (2012). Influence of curing conditions on the mechanical performance of concrete containing recycled plastic aggregate. Constr. Build. Mater..

[32] Amin M.N., Khan K., Saleem M.U., Khurram N., Niazi M.U.K. (2017). Aging and curing temperature effects on compressive strength of mortar containing lime stone quarry dust and industrial granite sludge. Materials.

[33] Ismail Z.Z., AL-Hashmi E.A. (2008). Use of waste plastic in concrete mixture as aggregate replacement. Waste Manag..

[34] Mustafa M.A.-T., Hanafi I., Mahmoud R., Tayeh B. (2019). Effect of partial replacement of sand by plastic waste on impact resistance of concrete: Experiment and simulation. Structures.

[35] Rai B., Rushad S.T., Kr B., Duggal S.K. (2012). Study of waste plastic mix concrete with plasticizer. Int. Sch. Res. Not..

[36] Ghernouti Y., Rabehi B., Safi B., Chaid R. (2011). Use of recycled plastic bag waste in the concrete. J. Int. Sci. Publ. Mater. Methods Technol..

[37] Akinyele J.O., Ajede A. (2018). The use of granulated plastic waste in structural concrete. Afr. J. Sci. Technol. Innov. Dev..

[38] Saxena R., Siddique S., Gupta T., Sharma R.K., Chaudhary S. (2018). Impact resistance and energy absorption capacity of concrete containing plastic waste. Constr. Build. Mater..

[39] Yu P., Manalo A., Ferdous W., Abousnina R., Salih C., Heyer T., Schubel P. (2021). Investigation on the physical, mechanical and microstructural properties of epoxy polymer matrix with crumb rubber and short fibres for composite railway sleepers. Constr. Build. Mater..

[40] Siddika A., Hajimohammadi A., Ferdous W., Sahajwalla V. (2021). Roles of waste glass and the effect of process parameters on the properties of sustainable cement and geopolymer concrete—A state-of-the-art review. Polymers.

[41] Abousnina R., Alsalmi H.I., Manalo A., Allister R.L., Alajarmeh O., Ferdous W., Jlassi K. (2021). Effect of short fibres in the mechanical properties of geopolymer mortar containing oil-contaminated sand. Polymers.

[42] Wongkvanklom A., Posi P., Homwuttiwong S., Sata V., Wongsa A., Tanangteerapong D., Chindaprasirt P. (2019). Lightweight geopolymer concrete containing recycled plastic beads. Key Eng. Mater..

[43] Alqahtani F.K., Ghataora G., Dirar S., Khan M.I., Zafar I. (2018). Experimental study to investigate the engineering and durability performance of concrete using synthetic aggregates. Constr. Build. Mater..

[44] Jacob-Vaillancourt C., Sorelli L. (2018). Characterization of concrete composites with recycled plastic aggregates from postconsumer material streams. Constr. Build. Mater..

[45] Aldahdooh M., Jamrah A., Alnuaimi A., Martini M., Ahmed M. (2018). Influence of various plastics-waste aggregates on properties of normal concrete. J. Build. Eng..

